# Morphology Adjustment and Optimization of CuS as Enzyme Mimics for the High Efficient Colorimetric Determination of Cr(VI) in Water

**DOI:** 10.3390/nano12122087

**Published:** 2022-06-17

**Authors:** Xinman Tu, Linhong Ge, Lamei Deng, Li Zhang

**Affiliations:** 1 Key Laboratory of Jiangxi Province for Persistent Pollutants Control and Resources Recycle, Nanchang Hangkong University, Nanchang 330063, China; tuxinman@nchu.edu.cn (X.T.); glhong2022@163.com (L.G.); denglamei0617@163.com (L.D.); 2 National-Local Joint Engineering Research Center of Heavy Metals Pollutants Control and Resource Utilization, Nanchang Hangkong University, Nanchang 330063, China

**Keywords:** CuS, morphology adjustment, solvent ratio, Cr(VI), colorimetric detection

## Abstract

Metal sulfide is often utilized as a catalyzed material to form colorimetric response system for some heavy metal detection. While the aggregation effect and conventional morphology limited the catalyzed efficiency. Herein, a robust method based on morphology adjustment was proposed to improve the dispersibility and catalytic performance of CuS. The results demonstrated when the solvent ratio of ethylene glycol and dimethyl sulfoxide arrived at 3:1, it displayed an optimal structure which is like a patulous flower. Meanwhile, an optimal surface binding energy (ΔE) of 120.1 kcal/mol was obtained via theoretical calculation model. The flower-like structure caused a 2-fold increase in the catalytic level. Subsequently, the CuS was employed to make colorimetric detection of Cr(VI) in water. The assay results exhibited a linear range of the Cr(VI) from 60 to 340 nM, the limit of detection was 1.07 nM. In the practical tests for Qianhu lake water, the spiked recoveries were 93.6% and 104% with the RSD of 4.71% and 3.08%. Therefore, this CuS-based colorimetric method possesses a satisfactory application prospect for the Cr(VI) determination in water.

## 1. Introduction

The heavy metal pollution in the environment caused by industrial production is increasing, the contaminative soil and water seriously threaten human health [1]. As a typical one, the hexavalent chromium (Cr(VI)) has become an attention focus owing to the high toxicity and carcinogenicity [2,3,4]. Therefore, it is of great significance for environment monitoring to establish a rapid and sensitive determination method for Cr(VI). The common detection techniques are mainly based on spectrophotometer, atomic absorption, mass spectrometry and electrochemistry [5,6,7,8]. Whereas, those techniques required complex pre-treatment procedures and professional operation, which was difficult to meet the requirement of daily monitoring.

The colorimetric strategy provides a convenient and rapid detection approach for heavy metals, which can merely use the naked eye to make distinction [9,10]. The conventional colorimetric methods are mostly based on the redox between peroxidases and H_2_O_2_, the produced •OH can further make a color conversion with some medium like 3,3′,5,5′-tetramethylbenzidine (TMB) [11]. However, the chelation of metal ion to enzyme often leads to low detection efficiency. Meanwhile, the storage of the enzymes has also faced a challenge of high cost. To resolve the issues, some nanomaterials containing enzyme-like property are proposed [12,13,14]. They possess high stability and catalysis, and the influence of other metal ions on them is limited.

The common nanozymes include metal oxides and metal sulfides, such as Fe_3_O_4_ and CuS. They were proved to own peroxidase property, and widely utilized in sensor development, chemical analysis and environmental remediation [15,16]. However, as a functional monomer material, their catalysis capacity is limited. Meanwhile, the high specific surface energy of them faces a challenge of aggregate or precipitate in aqueous solution, which further impedes the catalytic activity [17]. To obtain a more efficient application, the nanozymes were cooperated with other functional groups to construct nanocomposites like MoS_2_@CoFe_2_O_4_, CuS/rGO and CuS-BSA [18,19,20]. Although these nanocomposites achieved an improvement of the catalysis efficiency, the aggregate effect remained, and the multiple preparation procedures as well as low productivity became a novel inconvenience. Inspired by aperture adjustment of metal-organic framework [21], adjusting the size and morphology of metal oxide or metal sulfide monomer to enhance specific surface area was proposed as a novel way to enhance catalysis performance.

**Scheme 1:**
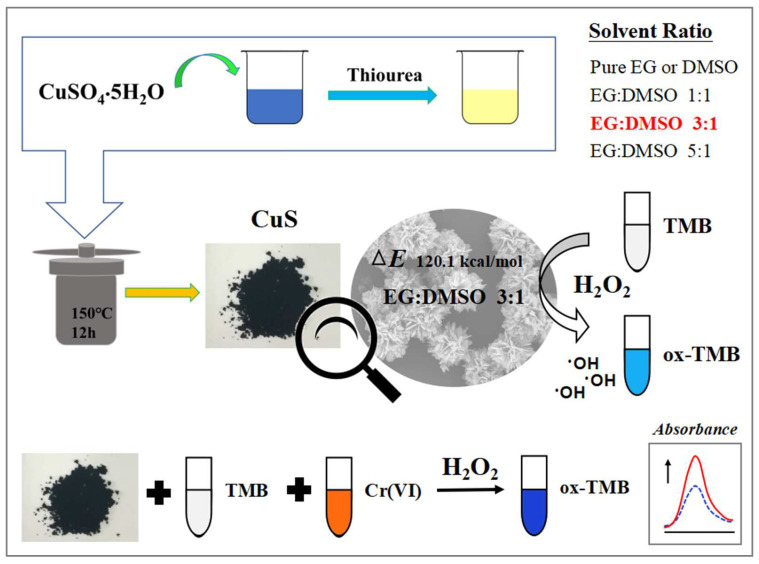
The synthesis of CuS and the use for colorimetric determination of Cr(VI).

Herein, CuS was utilized as a case to adjust the morphology to improve the catalysis performance, and it was then employed to detect Cr(VI) in water. By adjusting the solvent ratio in the one-pot solvothermal interaction procedure, the morphology feature of the synthesized CuS was conversed, which further improved the dispersion and electron transfer rate. Subsequently, this CuS was applied to make colorimetric determination of Cr(VI) with H_2_O_2_ and TMB (Scheme 1). Under the synergistic effect of the optimized CuS and target Cr(VI), the degree and velocity of the color conversion that sensitive to the amount of Cr(VI) was obviously increased. In comparison to other colorimetric methods based on nanozymes, this work focuses on the adjustment of material morphology, which avoids complicated material preparation process, and supports a more simplified and efficient detection of Cr(VI).

## 2. Materials and Methods

### 2.1. Reagents and Apparatus

The Cu(NO_3_)_2_•3H_2_O, sodium acetate, acetic acid, potassium dichromate, H_2_O_2_ and dimethyl sulfoxide (DMSO) were purchased from Xilong Chemical Co. Ltd. (Lanzhou, China). The thiourea, ethanol, 3,3′,5,5′-tetramethylbenzidine (TMB), terephthalic acid, Na_2_SO_4_•10H_2_O, ZnSO_4_•H_2_O and FeN_3_O_9_•9H_2_O were purchased from Aladdin Bio-Chem Technology Co. Ltd. (Shanghai, China). The ethylene glycol (EG), NiSO_4_ and cobaltous acetate were purchased from Macklin Bio-Chem Technology Co. Ltd. (Shanghai, China). These reagents used in assays are analytical grades. The ultrapure water was obtained through HFB-10 purifier (Shanghai, China).

The fluorescence spectra assays were performed to prove catalytic mechanism, which use F-7000 fluorescence spectrophotometer (Hitachi, Tokyo, Japan) under the voltage of 700 V with the Ex/Em slit of 5.0/5.0 nm. The UV-Vis spectrum assays were performed to exhibit the level of the colorimetric conversions, which use UV-3900H ultraviolet spectrophotometry (Hitachi, Japan). For the materials characterizations, the surface diffraction assays were performed by Ultima IV X-ray Powder Diffractometer (XRD) (Bruker, Karlsruhe, Germany), the size and morphology were characterized by S-4800 field emission scanning electron microscope (SEM) (Hitachi, Japan), and the energy dispersive spectrum (EDS) analysis was performed by Axis UtraDLD X-ray energy spectrometer (Shimadu, Kyoto, Japan).

### 2.2. Synthesis of CuS

The synthesis procedure was based on a mixed solvothermal method [22]. The 0.242 g of Cu(NO_3_)_2_•3H_2_O was scattered into a 56 mL of mixture contained EG and DMSO with a volume ratio of 3:1. After stirring for 0.5 h, the 0.228 g of thiourea was further added into the mixture to continue stirring for 0.5 h. The mixture was then transferred into reactor to incubate at 150 °C for 12 h. After cooling it to room temperature, the product was taken to make centrifugation at 10,000 r/min for 5 min, followed by washing using distilled water and ethanol for three times, respectively. After drying at 70 °C, the target material CuS was obtained. Subsequently, different solvent ratios of the EG and DMSO were employed to synthesize the CuS, while the other synthesis procedures remained.

### 2.3. Activity Analysis

Firstly, the CuS nanomaterials prepared by different solvent ratios were scattered into water, respectively. Subsequently, a 2 mL of 0.20 M acetate-acetate buffer solution (pH 4.0) contained the 60 µg/mL CuS, 0.80 mM TMB and 0.12 mM H_2_O_2_ was prepared. It was then incubated into water at 37 °C for 20 min. The colorimetric conversions were observed by the naked eye, and the colorimetric level was measured by the UV/V is spectrophotometer (λ = 652 nm). Moreover, under the same interaction condition and procedure, different concentrations of H_2_O_2_ were prepared, and the produced colorimetric signals were recorded to calculate the *K*_m_ of them. Similarly, different concentrations of TMB were then prepared, and the colorimetric results were recorded to calculate the relevant *K*_m_.

### 2.4. Investigation of Catalytic Mechanism

Firstly, a 2 mL of 0.20 M acetate-acetate buffer solution (pH 4.0) contained 0.10 mM fluorescent substrate terephthalic acid and 40 µg/mL CuS was prepared. This solution was incubated at 25 °C for 30 min to record the fluorescence intensity by the spectrophotometer. Subsequently, the 40 µg/mL CuS was replaced by 0.20 mM H_2_O_2_ and 80 nM Cr(VI) to perform the fluorescence assays, respectively. Furthermore, the mixtures of the CuS + H_2_O_2_, Cr(VI) + H_2_O_2_, CuS + Cr(VI) + H_2_O_2_ were respectively added into the acetate-acetate buffer solution with the terephthalic acid to make the fluorescence measurements under the same condition.

### 2.5. Determination of Cr(VI)

Multiple 2 mL of 0.20 M acetate-acetate buffer solution (pH 4.0) contained 100 μg/mL CuS, 0.80 mM TMB, 0.16 mM H_2_O_2_ and different concentrations of Cr(VI) were prepared. After incubation at 25 °C for 10 min, the colorimetric intensities were measured by the UV/Vis spectrophotometer (λ = 652 nm), and the distinctions of them to that of the contrast system containing CuS, TMB and H_2_O_2_ were recorded to make the calibration plot. In the real sample test, the water taken from Qianhu lake was filtrated by a polyethersulfone (PES) membrane (0.22 µm pore diameter). The 2 mL of the water was added into 2 mL of 0.40 M acetate-acetate buffer solution (pH 4.0) containing 200 μg/mL CuS, 1.6 mM TMB, 0.32 mM H_2_O_2_. Under the same procedure exhibited above, the amount of target Cr(VI) was measured. In the spiked assays, according to the measured Cr(VI) level, different concentrations of Cr(VI) were added into the water sample before pre-treatment. After filtration, the measurements were performed with the same procedure to calculate the recoveries.

## 3. Results and Discussion

### 3.1. Material Characterization

**Figure 1:**
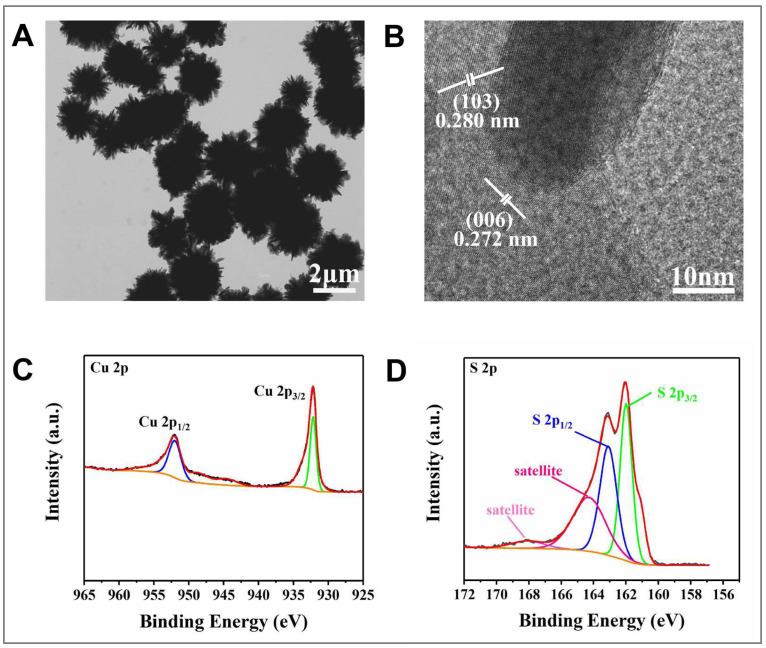
The TEM (**A**) and high resolution TEM (**B**) graph of the prepared CuS nanomaterial; The high resolution XPS spectrum of Cu 2p (**C**) and S 2p (**D**).

The CuS was synthesized by a solvothermal method. To prove the construction of it, a TEM assay was first performed. As the results displayed in Figure 1A, the product presented a flower-like structure with the size of 1 µm (inset). By calibrating the lattice fringes, the spacing was confirmed as 0.280 and 0.272 nm, which belonged to the (103) and (006) crystal planes of CuS, respectively (Figure 1B) [23]. To investigate the ingredient, the energy spectrum point and flat scans were made. As the results shown in Figure S1, the product contained the elements of Cu and S. These results demonstrated that the synthesized product is CuS. To analyze the valence states of the contained Cu and S, a high-resolution XPS assay was introduced. Two feature peaks of Cu 2p were observed at 932.3 eV (Cu 2p3/2) and 952.3 eV (Cu 2p1/2) in binding energy graph (Figure 1C). This proves the Cu existing in a bivalent form. Similarly, as Figure 1D showed, two feature peaks of S 2p were observed at 162.1 eV (S 2p3/2) and 163.2 eV (S 2p1/2). Meanwhile, two satellite peaks of S 2p1/2 were additionally found at 164.9 and 168.1 eV. This indicates the S is bivalent [23].

### 3.2. Morphology Adjustment and Optimization

**Figure 2:**
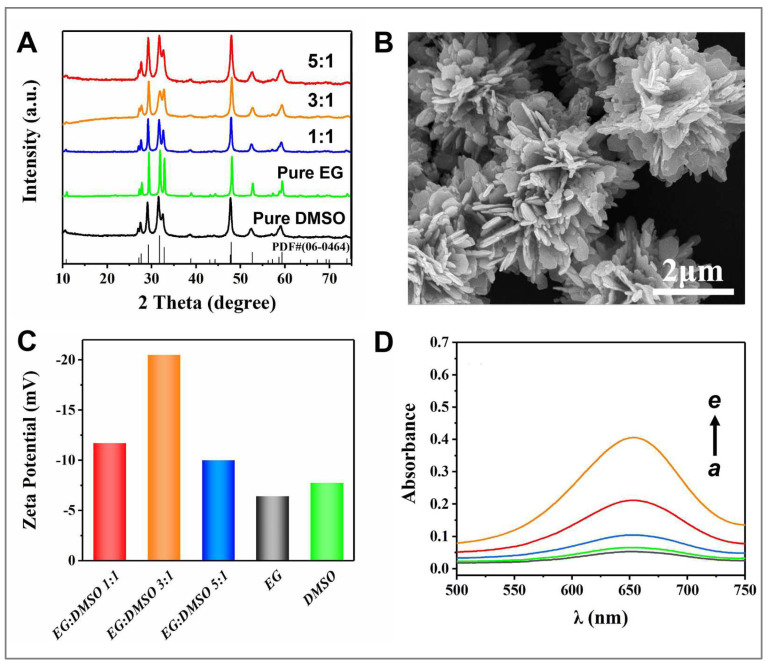
(**A**) The XRD patterns of the CuS synthesized by different solvent proportions; (**B**) The SEM image of the CuS synthesized by EG and DMSO with solvent ratio of 3:1; (**C**) The zeta potential of the CuS synthesized by different solvent proportions; (**D**) The absorbance signals produced by the CuS synthesized via different solvent proportions (line a–e: pure EG, pure DMSO, 5:1, 1:1, 3:1).

After confirmation of the synthesized product, an adjustment for the CuS morphology was performed via regulating the solvent ratios of the EG and DMSO. The solvents containing EG and DMSO with the ratios of 1:1, 3:1 and 5:1, as well as pure EG or DMSO, were utilized in the assays. The XRD graphs (Figure 2A) illustrate that the diffraction peaks of all the products matched those of CuS standard spectrum, confirming the synthesis results of the CuS. Subsequently, the SEM assays were performed to investigate surface morphology. As the graph showed in Figure 2B, the CuS prepared using the EG and DMSO with the ratios of 3:1 displayed a patulous flower-like structure with uniform size, suggesting a large specific surface area. However, the CuS prepared by the solvents with the ratios of 1:1 and 5:1 exhibited compact decussate structure (Figure S2A) and multihole globular structure (Figure S2B), respectively. Meanwhile, that prepared by pure EG or DMSO solvent orderly displayed a multihole globular morphology (Figure S2C) and a compact ring-bundle morphology (Figure S2D).

To further investigate the influence of solvent ratio on the morphology feature, a theoretical calculation was performed. The surface structures of the CuS made by different solvent ratios were constructed according to the crystal face of CuS (103) in relevant XRD graphs. The Sorption and Dmol3 were utilized to confirm the sorbent location and surface binding energy (Δ*E*), respectively. In the procedure, the EG and DMSO were fixed with ratios of 1:1, 3:1 and 5:1, respectively. The generalized gradient approximation developed by Perdew, Burke and Ernzerhof. The real-space global obital cut-off was 4.4 Å, and the convergence threshold parameters for the optimization was 10^−5^ Ha, with the gradient of 2 × 10^−3^ Ha/Å and the displacement of 5 × 10^−3^ Å. The Δ*E* was calculated by an equation as followed.

Δ*E* = *E*(AnBm) − n*E*(A) − m*E*(B)

where the *E*(AnBm) is the total free energy of CuS, EG and DMSO, *E*(A) is that of CuS, and *E*(B) is that of EG and DMSO. As Figure S3 showed, the Δ*E* of the CuS with 3:1 solvent ratio was the maximum (120.1 kcal/mol), and the free energy (*E*) was moderate. This caused a mutual pull effect during the synthesis procedure, and formed an exoteric construction. However, when the EG level was higher, both the Δ*E* and *E* declined. While the EG level was equal to that of DMSO, the *E* was increased, and the Δ*E* was reduced. Therefore, the EG amount was required to be controlled as 3-fold of the DMSO, which can maintain the *E* and Δ*E* at appropriate values.

Subsequently, a zeta potential test was made to compare the properties of the prepared CuS. As the results shown in Figure 2C, all the CuS present negative potential, and the highest one is that using 3:1 solvent ratio of the EG and DMSO, which is nearly 2-fold to the others, suggesting an optimal stability and dispersibility of this CuS in water. The following assays results (Figure S4) showed that the CuS made by pure EG or DMSO was aggregated and deposited in water, whereas those made by the mixed solvent of EG and DMSO were dispersed, and this dispersion trend fits that of the zeta potential level. Favourable dispersion commonly signifies favourable catalytic efficiency. Therefore, a colorimetric assay based on the peroxidase property of the CuS was then performed to investigate catalytic function. The 2 mL of 0.20 M acetate-acetate buffer solution (pH 4.0) containing 0.80 mM TMB, 0.12 mM H_2_O_2_ and 60 µg/mL different types of CuS were prepared. After incubation in water at 37 °C for 20 min, the produced color conversions were respectively recorded as absorbance values. Figure 2D showed that the absorbance produced by the CuS making of EG and DMSO (3:1) is obviously higher than the others, indicating the catalytic property of it is optimal.

### 3.3. Activity Analysis

**Figure 3:**
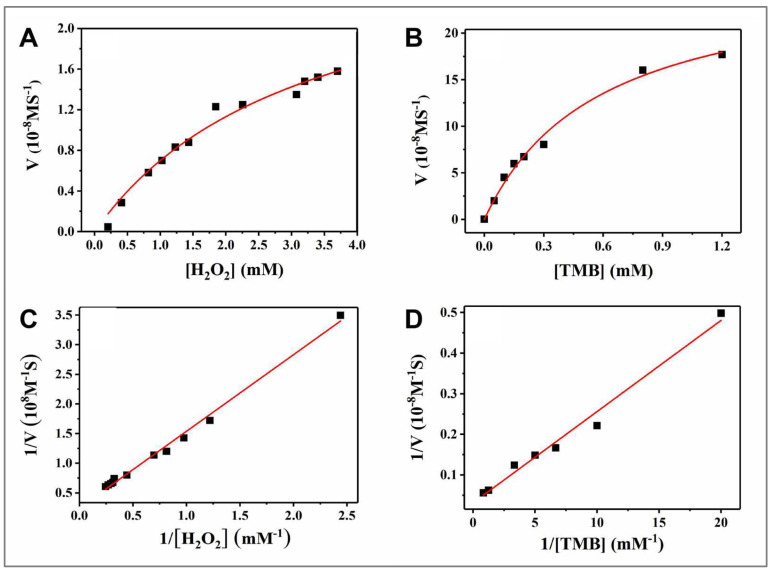
The kinetic process of 60 µg/mL CuS to 0.8 mM TMB and different concentrations of H_2_O_2_ (**A**), as well as to 0.12 mM H_2_O_2_ and different concentrations of TMB (**B**) at 37 °C for 20 min; The relevant Lineweaver-Burk plots for the H_2_O_2_ (**C**) and the TMB (**D**).

After confirmation of the optimal CuS type, the kinetic activity of it was further analyzed. The kinetic constant *K*_m_ which expresses the affinity of the catalyst to substrate was measured. Commonly, a lower *K*_m_ value reflects a higher interaction affinity [24]. In the catalytic interaction system, the substrates H_2_O_2_ and TMB containing different concentrations were respectively employed to perform the colorimetric assays. The color changes were recorded as the absorbance values, and the following calculation is based on the equation below:

$$V=V_{\max}\frac{\left[S\right]}{K_{m}+[S]}$$

where *V* is the catalytic rate; *V*_max_ is the maximum rate conversion, obtaining from the catalytic sites when the enzyme is saturated with the substrate; [*S*] is the initial substrate concentration. To calculate conveniently, the equation was further conversed as followed:

$$\frac{1}{V}=\frac{K_{m}+[S]}{V_{\max}[S]}=\frac{K_{m}}{V_{\max}}\frac{1}{\left[S\right]}+\frac{1}{V_{\max}}$$

where the *K*_m_/*V*_max_ and 1/*V*_max_ are reflected by the linear slope and intercept. Figure 3A,B illustrated the catalytic rates under different concentrations of H_2_O_2_ and TMB, respectively. According to the formed Lineweaver-Burk plots (Figure 3C,D), the *K*_m_ values for the H_2_O_2_ and TMB were 3.219 and 0.534 mM, respectively. Additionally, the *V*_max_ for the H_2_O_2_ and TMB were 2.90 × 10^−8^ and 26.65 × 10^−8^ Ms^−1^. In comparison to other natural peroxidase or nanomaterials, this CuS nanomaterial has certain benefits on interaction affinity and efficiency (Table S1). Relying on the favourable peroxidase property, the CuS was then utilized to make a determination of H_2_O_2_. As the results shown in Figure S5, a linear range from 6 to 120 µM was obtained with an equation of *A* = 0.0028 C_H_2_O_2__ + 0.1445 (R^2^ = 0.9843), and the detection limit was calculated as 3.2 µM (3σ/S), suggesting it possesses a good signal response to H_2_O_2_.

### 3.4. Condition Optimization

To investigate the practicability of the CuS, it was utilized to detect Cr(VI), and the detection conditions including CuS dosage, pH value and interaction temperature were orderly optimized. As the assay results showed, an increased amount of CuS led to a continuous raising of the catalytic efficiency, while the signal turned down after the CuS concentration achieved 100 µg/mL (Figure S6A). This is because the overload level of CuS causes an unspecific accumulation, and inhibits the catalysis procedure. In addition, it showed an inverted V-shaped trend between the pH values and the relative activity, and the top point correspond to a pH of 4.0 (Figure S6B). The following assays were performed using 100 µg/mL CuS in the buffer (pH 4.0) at different temperatures. As Figure S6C showed, from 20 °C to 25 °C, the relative activity of CuS enhanced dramatically, while it gradually declined under the following temperatures. Therefore, the optimal temperature was confirmed as 25 °C.

### 3.5. Determination Property Assessment

**Figure 4:**
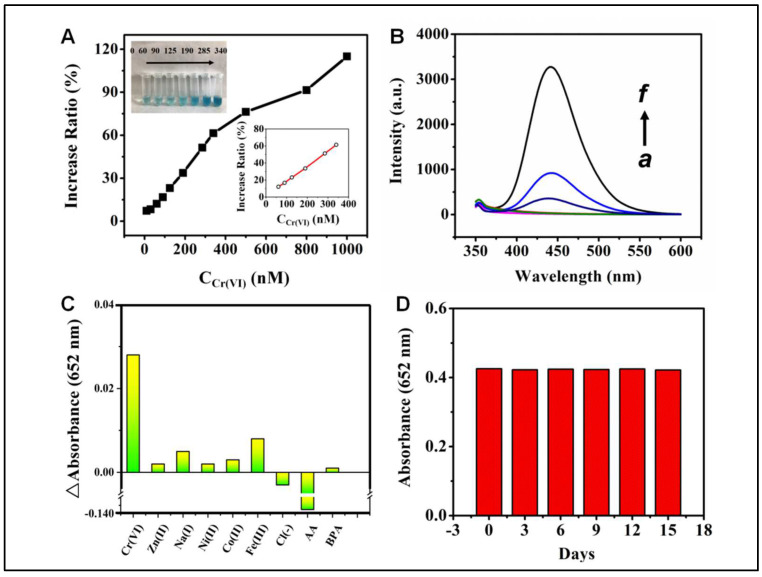
(**A**) The point plot relating to the increased ratio of the absorbance and the concentrations of the Cr(VI), and the linear relationship plot of the Cr(VI) in the concentration range of 60–340 nM (nether inset), as well as the colorimetric assay results adding 0, 60, 90, 125, 190, 285 and 340 nM Cr(VI) (above inset); (**B**) The fluorescence signals produced by CuS + TA (line a), H_2_O_2_ + TA (line b), Cr(VI) + TA (line c), Cr(VI) + H_2_O_2_ + TA (line d), CuS + H_2_O_2_ + TA (line e) and CuS + Cr(VI) + H_2_O_2_ + TA (line f); (**C**) Selectivity assay results for the CuS based colorimetric detection method; (**D**) Stability assay results for the CuS storing to avoid light at room temperature for 0, 3, 6, 9, 12 and 15 days.

Under the optimal detection condition, the Cr(VI) determination was performed. Different amounts of the target Cr(VI) were added into the 2 mL of 0.20 M acetate-acetate buffer solution (pH 4.0) containing 100 μg/mL CuS, 0.80 mM TMB and 0.16 mM H_2_O_2_ to incubate at 25 °C for 10 min. The signal distinctions of before and after addition of Cr(VI) were recorded and made a relevant point plot (Figure 4A), it exhibited a linearity from 60 to 340 nM (nether inset) with an equation of Δ*A* = 0.176C_Cr(VI)_ + 0.994, the regression coefficient (R^2^) was 0.9992, and the detection limit was 1.07 nM (3σ/S). According to the requirement of the world health organization (WHO), the Cr(VI) in the water should be under 50 μg/L (–960 nM). This indicates the strategy applicability was satisfactory. Meanwhile, in comparison of other colorimetric determination methods for Cr(VI) [25,26,27,28], the detection performances of this strategy possess certain benefits (Table 1). From the corresponding colorimetric results (0, 60, 90, 125, 190, 285 and 340 nM), the color distinction was obvious (Figure 4A, inset), which supports the naked eye measurements.

To prove the reliability of the colorimetric results, a verification test based on fluorescence analysis was performed using terephthalic acid (TA) which is able to produce fluorescence signal with •OH. Figure 4B illustrated little signal when the Cr(VI), CuS or H_2_O_2_ solely incubated with TA. However, a low signal was presented when the Cr(VI) and H_2_O_2_ mixed with TA (line d), and a higher signal was observed when the CuS and H_2_O_2_ mixed with TA (line e). The results suggested both the Cr(VI) and CuS can produce •OH with H_2_O_2_. Meanwhile, an obvious signal increase was found from the mixture of Cr(VI), CuS, H_2_O_2_ and TA (line f). This suggested an induced reaction between the Cr(VI) and CuS [25,29]. Associating to the colorimetric response status, an interaction procedure was deduced as followed. Generally, the rapid cyclical catalytic interaction between CuS and H_2_O_2_ improved the reaction efficiency of Cr(VI) and H_2_O_2_ by providing more H^+^. The Cr_2_O_7_^2−^ dissolved in water can be converted to be CrO_4_^2−^ and H^+^. The half-reaction potential (*φ*) of CrO_4_^2−^ is −0.12 V, suggesting a low efficient interaction procedure. However, once more H^+^ presented, the Cr_2_O_7_^2−^ can be reproduced via the common ion effect. The *φ* value of Cr_2_O_7_^2−^ is 1.33 V, which is beneficial for the redox with H_2_O_2_. It is worth noticing that although the oxidized form of the CuS owns oxidability, the TMB prefers to be oxidized by the produced •OH. This is because the *φ* value of •OH arrived at 2.80 V, which is obviously higher than that of the high valence state of Cu, indicating the •OH possesses stronger oxidation properties.

1.1. Cu(II) + H_2_O_2_ → Cu(III) + •OH + OH^−^
1.2. Cu(III) + H_2_O_2_ → Cu(II) + •OOH + H^+^
2.1. Cr(VI) + H_2_O_2_ + H^+^ → Cr(III) + H_2_O + O_2_
2.2. Cr(III) + H_2_O_2_ → Cr(VI) + •OH + OH^−^
3. OH + TMB (colorless) → oxTMB (blue color)

Subsequently, a selectivity assay was performed using Cr^6+^, Zn^2+^, Na^+^, Ni^2+^, Co^2+^, Fe^3+^, Cl^−^, bisphenol A (BPA) and ascorbic acid with the equal amount. As the result shown in Figure 4C, merely Cr(VI) provoked an obvious forward signal distinction, indicating a dependable selectivity of this detection strategy. It is interesting that ascorbic acid caused a large reversed signal response owing to the strong reducibility. Even so, ascorbic acid commonly presences in biological samples, the possibility of ascorbic acid existing in lakes or rivers is low. Therefore, in a given sample, the influence of ascorbic acid on this strategy is limited. Meanwhile, the measurable signal response to ascorbic acid signifies a potential application of this strategy in biological sample assays. Additionally, a stability assay was performed. The CuS was then stored to avoid light at room temperature for 3, 6, 9, 12 and 15 days. The ones were then orderly used to perform the colorimetric assays. From the result shown in Figure 4D, the produced colorimetric signals remained at the same level, proving the favourable stability of the prepared CuS.

### 3.6. Practical Tests

To assess the practicability of the proposed colorimetric method based on the CuS, a certain amount of Qianhu lake water was taken and utilized for the assay as the practical sample. The water sample was stored at room temperature for 30 min, and the supernatant liquid was filtrated by a polyethersulfone (PES) membrane with 0.22 µm pore diameter. The 2 mL of the filtrated water sample was added into equal volume of 0.4 M acetate-acetate buffer solution (pH 4.0) containing 200 μg/mL CuS, 1.6 mM TMB, 0.32 mM H_2_O_2_. After incubation at 25 °C for 20 min, the color conversion level was recognized as the absorbance signal to measure the Cr(VI) amount. Subsequently, the Cr(VI) solvents were added into the original water sample, and the final concentrations of the spiked Cr(VI) were 100 and 200 nM. After the same pre-treatment procedure, the spiked samples were taken to the assay under the same condition. Table 2 illustrated the assay results. The Cr(VI) in the water sample was undetected, and the spiked recoveries were 93.6% and 104% with the RSD of 4.71% and 3.08%, respectively. Similarly, the contrast method based on 1,5-diphenylcarbazide spectrophotometric method demonstrated the detection result is reliable, which further proves the practicability of the detection method.

## 4. Conclusions

This work mainly focuses on the improvement of the CuS catalytic performance by adjusting and optimizing the intrinsic morphology. The assays indicated when the solvent ratio of EG and DMSO arrived at 3:1, the specific surface area, dispersibility and stability of the prepared CuS were increased obviously. This result was further proved via theoretical simulation calculation. Based on the favourable peroxidase property, the CuS was then employed to make Cr(VI) detection via a simple colorimetric approach. Under the synergistic effect of the CuS and target Cr(VI), the production level of ox-TMB was enhanced obviously, which improved the degree and velocity of the color conversion that sensitive to the target amount, and obtained a sensitive detection limit of 1.07 nM. Meanwhile, the satisfactory spiked recovery results in the practical tests suggest a prospect application of this strategy in environmental pollutants monitoring.

## Figures and Tables

**Table 1:** The comparison of this CuS based strategy and other nanomaterials based methods for colorimetric determination of Cr(VI).

Material	Line Range (nM)	Detection Limit (nM)	Refs.
CuS	60–340	1.07	This work
CuS-BSA	0–100 and 1 × 10^3^–2 × 10^4^	50 and 6.8 × 10^3^	[25]
frGO-Cu	0–200	67	[26]
PVP-AgNPs	1 × 10^2^–2.4 × 10^3^	34	[27]
Fe_3_O_4_@MFR	50–3000	38	[28]

**Table 2:** The detection of Cr(VI) in Qianhu lake water and the spiked assay results (*n* = 3).

Sample	Spiked (nM)	This Method			Contrast Method (1,5-Diphenylcarbazide Spectrophotometry)		
		Measured (nM)	Recovery (%)	RSD (%)	Measured (nM)	Recovery (%)	RSD (%)
Qianhu Lake Water	0	ND	/	/	ND	/	/
	100	93.6	93.6	4.71	96.5	96.5	3.19
	200	208	104	3.08	195	97.5	2.26

ND = not detected.

## Data Availability

Not applicable.

## Supplementary Materials

The following supporting information can be downloaded at: https://www.mdpi.com/article/10.3390/nano12122087/s1. Figure S1: (A) The EDS spectra of the synthesized CuS; (B)-(C) The elemental mappings of the synthesized CuS. Figure S2: SEM images of the synthesized CuS by EG and DMSO with the solvent ratios of 1:1 (A), 5:1 (B), as well as pure EG (C) and DMSO (D). Figure S3: The results of the theoretical simulation calculation for the E and ∆E of the CuS prepared by different solvent ratios. Figure S4: The dispersion of the synthesized CuS by EG and DMSO with the solvent ratios of 3:1 (a), 1:1 (b), 5:1 (c), as well as pure EG (d) and pure DMSO (e). Figure S5: The point plot relating to the absorbance signals and the concentrations of H_2_O_2_, and the linear relationship plot of H_2_O_2_ in the concentration range of 6~120 µM (nether inset), as well as the colorimetric assay results of H_2_O_2_ (above inset), the target concentrations from left to right are 0, 75, 125, 225 and 350 µM. Figure S6: The optimization of the CuS concentration (A), buffer pH (B) and interaction temperature (C). Table S1: The comparison of activity parameters of CuS and other nanoenzymes. References [25,30,31,32] are cited in the Supplementary Materials.
