# Supplementary material for: Morphology Adjustment and Optimization of CuS as Enzyme Mimics for the High Efficient Colorimetric Determination of Cr(VI) in Water
Source: Nanomaterials (Basel). 2022 Jun 17;12(12):2087. doi: 10.3390/nano12122087 (PMC9231408; doi:10.3390/nano12122087)
Supplement: Supplementary file 1 [file nanomaterials-12-02087-s001.zip › nanomaterials-1743303-supplementary.pdf]

<u>Supplementary Information</u>

Morphology Adjustment and Optimization of CuS as Enzyme Mimics for the High Efficient Colorimetric Determination of Cr(VI) in Water

Xinman Tu [1,2], Linhong Ge [1,2], Lamei Deng [1,2] and Li Zhang [1,2,*]

1 Key Laboratory of Jiangxi Province for Persistent Pollutants Control and Resources Recycle, Nanchang Hangkong University, Nanchang 330063, China; tuxinman@nchu.edu.cn (X.T.); glhong2022@163.com (L.G.); denglamei0617@163.com (L.D.)

2 National-Local Joint Engineering Research Center of Heavy Metals Pollutants Control and Resource
Utilization, Nanchang Hangkong University, Nanchang 330063, China

* Correspondence: zhangli@nchu.edu.cn

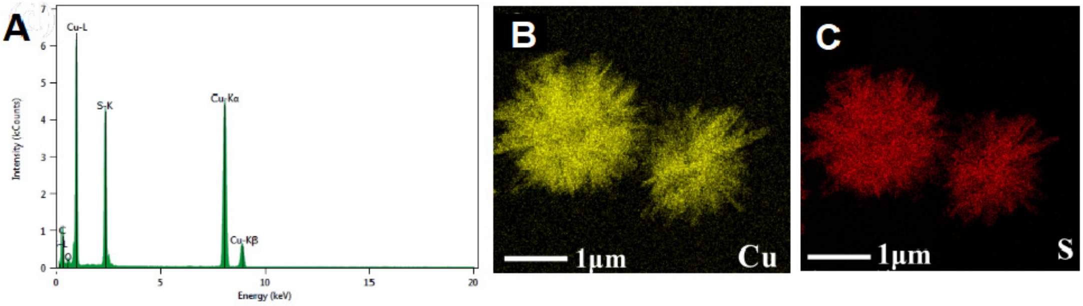

Figure S1. (A) The EDS spectra of the synthesized CuS; (B,C) The elemental mappings of the synthesized CuS.

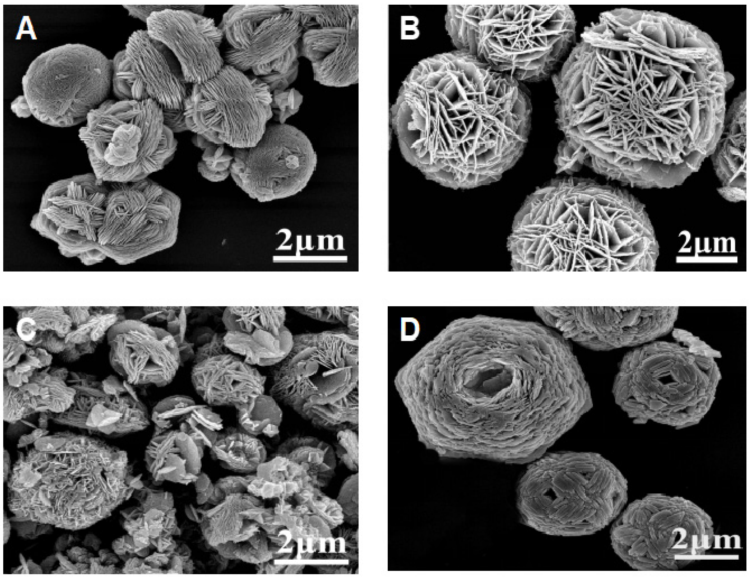

Figure S2. SEM images of the synthesized CuS by EG and DMSO with the solvent ratios of 1:1 (A), 5:1 (B), as well as pure EG (C) and DMSO (D).

		E	ΔE
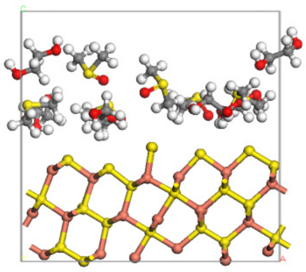	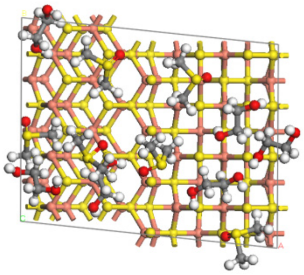	EG : DMSO (1:1)<br>-153069.18072 Ha	EG : DMSO (1:1)<br>-73.4 kcal/mol
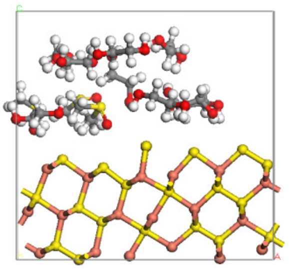	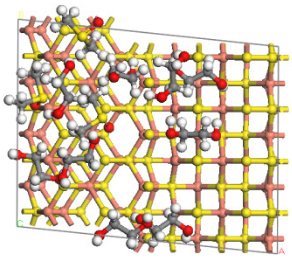	EG : DMSO (3:1)<br>-152100.64438Ha	EG : DMSO (3:1)<br>-120.1 kcal/mol
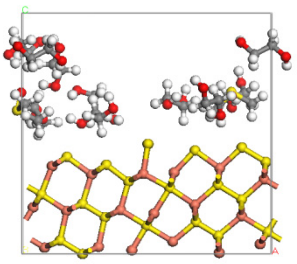	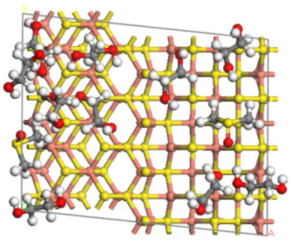	EG : DMSO (5:1)<br>-151777.74974Ha	EG : DMSO (5:1)<br>-104.8 kcal/mol

Figure S3. The results of the theoretical simulation calculation for the E and ΔE of the CuS prepared by different solvent ratios.

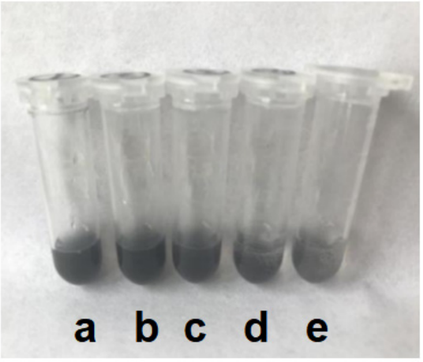

Figure S4. The dispersion of the synthesized CuS by EG and DMSO with the solvent ratios of 3:1 (a), 1:1 (b), 5:1 (c), as well as pure EG (d) and pure DMSO (e).

Table S1. The comparison of activity parameters of CuS and other nanoenzymes.

Catalysts	Substrates	K_m (mM)	V_m (10^{-8} MS^{-1})	Ref.
CuS	H_2O_2	3.219	2.90	This work
CuS	TMB	0.534	26.65	This work
HRP	H_2O_2	3.70	8.71	[30]
HRP	TMB	0.434	10	[30]
Cu NCs	H_2O_2	29.16	4.22	[31]
Cu NCs	TMB	0.648	5.96	[31]
Co_3O_4	H_2O_2	29.20	1.35	[32]
Co_3O_4	TMB	0.324	97.8	[32]
CuS-BSA	H_2O_2	14	2.0	[25]
CuS-BSA	TMB	0.2	3.3	[25]

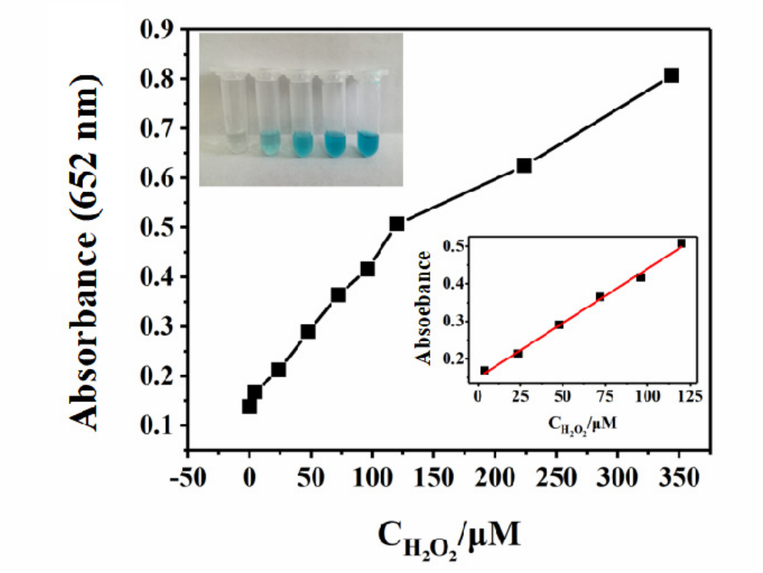

Figure S5. The point plot relating to the absorbance signals and the concentrations of H_2O_2, and the linear relationship plot of H_2O_2 in the concentration range of 6~120 μM (nether inset), as well as the colorimetric assay results of H_2O_2 (above inset), the target concentrations from left to right are 0, 75, 125, 225 and 350 μM.

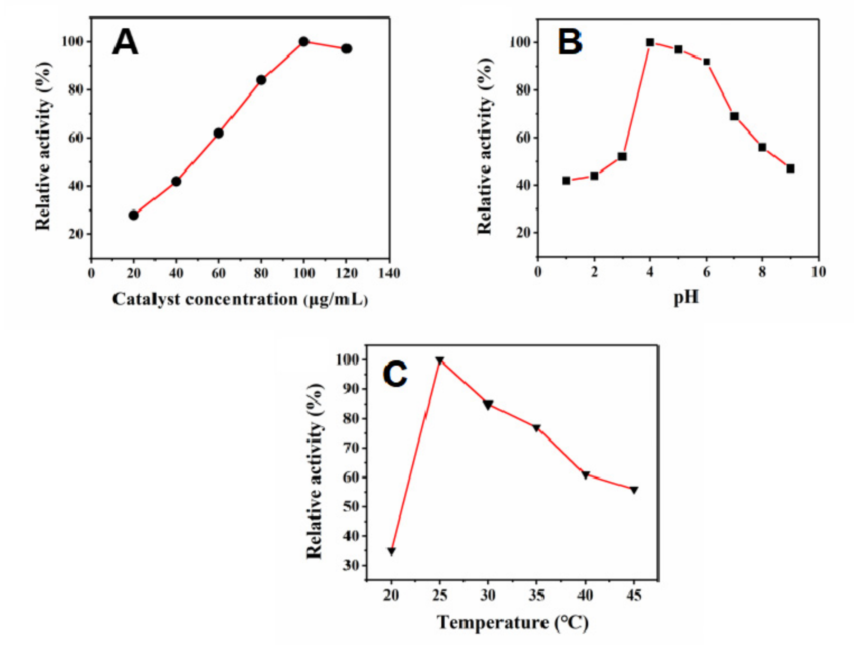

Figure S6. The optimization of the CuS concentration (A), buffer *p*H (B) and interaction temperature (C).

References

1. Swaidan, A.; Borthakur, P.; Boruah, K.P.; Das, R.M.; Barras, A.; Hamieh, S.; Toufaily, J.; Hamieh, T.; Szunerits, S.; Boukherroub, R. A facile preparation of CuS-BSA nanocomposite as enzyme mimics: Application for selective and sensitive sensing of Cr(VI) ions. *Sensor. Actuat. B Chem.* **2019**, *294*, 253–262. https://doi.org/10.1016/j.snb.2019.05.052.
2. Gao L., Zhuang J., Nie L., Zhang, J., Zhang, Y., Gu, N., Wang, T., Feng, J., Yang, D., Perrett, S., et al., Intrinsic peroxidase-like activity of ferromagnetic nanoparticles. *Nat. Nanotech*. **2007**, *2*, 577–583.
3. Hu, L., Yuan, Y., Zhang, L., Zhao, J., Majeed, S. and Xu, G., Copper nanoclusters as peroxidase mimetics and their applications to H_2O_2 and glucose detection. *Anal. Chim. Acta* **2013**, *762*, 83–86.
4. Liu, Q., Zhu, R., Du, H., Li, H., Yang, Y., Jia, Q. and Bian, B., Higher catalytic activity of porphyrin functionalized Co_3O_4 nanostructures for visual and colorimetric detection of H_2O_2 and glucose. *Mater. Sci. Eng. C* **2014**, *43*, 321–329.
